# Absolute Quantitative Lipidomics Reveals Differences in Lipid Compounds in the Blood of Trained and Untrained Yili Horses

**DOI:** 10.3390/vetsci12030255

**Published:** 2025-03-10

**Authors:** Tongliang Wang, Jun Meng, Jianwen Wang, Wanlu Ren, Xixi Yang, Wusiman Adina, Yike Bao, Yaqi Zeng, Xinkui Yao

**Affiliations:** 1 College of Animal Science, Xinjiang Agricultural University, Urumqi 830052, China; wtl13639911402@163.com (T.W.); junm86@xjau.edu.cn (J.M.); wjw1262022@126.com (J.W.); 13201295117@163.com (W.R.); xxyang2022@126.com (X.Y.); adina0901@126.com (W.A.); b18599635754@126.com (Y.B.); 2 Xinjiang Key Laboratory of Horse Breeding and Exercise Physiology, Urumqi 830052, China; 3 Horse Industry Research Institute, Xinjiang Agricultural University, Urumqi 830052, China

**Keywords:** performance horse training, lipidomics, echocardiography, remodeling, endurance, exercise regimens

## Abstract

The purpose of this study was to explore the relationship between blood lipid levels and the differences in cardiac structure and function of trained and untrained Yili horses as related to exercise performance. We utilized quantitative lipidomics technology to elucidate how the differences in lipid compounds in the blood influenced performance outcomes. Sixteen 18-month-old Yili horses were selected, ten of which received a 15-week training regimen, and six were kept as untrained controls. Cardiac structure and function were assessed by echocardiography, while plasma lipid metabolites were detected and identified by liquid chromatography–mass spectrometry. The results showed that key cardiac structural indices, such as left ventricular end-diastolic diameter, left ventricular end-systolic diameter, and left ventricular posterior wall thickness, were significantly greater in the trained group compared with the untrained group, indicating that exercise training promotes adaptive cardiac remodeling. Regarding lipid metabolites, significant differences were observed between the trained and untrained groups, with a total of 281 lipids identified—212 upregulated and 69 downregulated. These differentially expressed lipids were primarily enriched in pathways such as necroptosis, ether lipid metabolism, and sphingolipid signaling, which are associated with cell migration, survival, proliferation, and regulation of lipid metabolism. Further correlation analysis revealed that differences in certain lipids, such as PE (20:4_18:0), PC (17:0_18:1), and LPC subclasses, were significantly correlated with exercise-mediated cardiac structural and functional changes and exercise performance enhancement. These findings provide novel molecular insights into the effects of exercise training on cardiac structure and lipid metabolism in horses and can serve as a reference for training strategies and preserving cardiac health in performance horses.

## Simple Summary

This study aims to understand how training affects the heart structure and blood fat metabolism of the Yili horse, known for its athletic ability. Sixteen 18-month-old Yili horses participated in this study. Their heart size and function were measured using ultrasound, while their blood was analyzed to detect changes in fat-related substances. The results showed that training led to noticeable improvements in heart structure. Several types of fats in the blood also changed, mainly those linked to energy production, fat metabolism, and cell health. These findings offer valuable guidance for designing better training programs and ensuring the heart health of sport horses, benefiting both the equestrian industry and animal welfare.

## 1. Introduction

The structure and functionality of a horse’s heart can provide it with significant advantages in exercise capacity. The mass of a horse heart is proportionally much greater than that of other species, with an adult horse’s heart weighing on average 3.9 kg, while the heart of a racehorse can reach 6.3 kg, and that of a top-performing racehorse can weigh nearly 10 kg [1]. The heart rate of horses can increase from 35 beats per minute to 240 beats per minute, surpassing the capability of most species, including humans, and allowing them to significantly enhance cardiac output in a short time to meet the demands of athletic performance [2]. Researchers have measured heart size using echocardiography and found a significant positive correlation between left ventricular mass and maximal oxygen uptake [3].

Training is a key factor influencing heart size in horses. Studies have shown that high-intensity training leads to cardiac hypertrophy, thereby improving cardiac function. As training intensity increased, the heart mass of mature racehorses, particularly the left ventricle, increased significantly [4]. Compared to horses given lower intensity exercise, those receiving high-intensity training exhibited a larger LVID size [5]. However, excessive cardiac remodeling can also cause myocardial fibrosis, increasing the risk of arrhythmias [6].

The heart consumes a large amount of ATP daily to maintain basal metabolism and normal contraction, which is crucial for sustaining systemic blood pressure. The heart primarily relies on fatty acids as an energy source, with fatty acid oxidation providing most of the ATP required for cardiac contraction [7]. Under varying physiological conditions, the heart can switch metabolic substrates between fatty acids and glucose according to demand, maintaining a delicate balance in cardiac homeostasis through the uptake, storage, and utilization of high-energy fuels. Studies have shown that in patients suffering from heart failure and cardiomyopathy, cardiac energy substrates shift toward glucose utilization [8]. Lipid metabolism and the dynamic alteration of membrane lipid composition are critical for cardiac function. During heart development, an increase in the unsaturation levels of cardiolipin and phospholipids enhances mitochondrial function and energy metabolism [9].

Lipids are essential as both energy sources and basic structural components of the body. In horses, they are influenced by exercise type and intensity and are also vital substrates for skeletal muscle metabolism [10]. In Thoroughbred horses, changes in plasma concentrations of non-esterified fatty acids (NEFAs) and triglycerides before and after intense exercise indicate that lipid metabolism plays a crucial role in skeletal muscle energy production [11]. Lipidomics analysis using liquid chromatography–mass spectrometry (LC/MS) in Thoroughbred horses before and after training identified 933 differential lipids, with the signal intensity of 13 lipids significantly altered due to exercise [12]. A study of Arabian and part-Arabian endurance horses found that lipid biomarkers alone could not accurately determine where a horse would finish in a race, but highly correlated lipid metabolites were identified in the blood of the highest performing horses [13]. A follow-up experiment found that after finishing a race, horses exhibited increased glucose homeostasis, lipid metabolism, ketogenesis, ATP synthesis, and acetate production [14].

There have been numerous studies on exercise metabolomics of performance horses, consistently demonstrating significant differences in blood metabolites before and after exercise [15]. Significant correlations have also been observed between cardiac parameters and exercise performance. However, lipidomics research comparing the types and abundance of plasma lipids in trained vs. untrained horses is limited. This study conducted a comprehensive quantitative lipidomics analysis to examine changes in plasma lipid metabolites in Yili horses after three months of training. It explored the relationship between plasma lipid metabolites and cardiac structure and function and identified key lipid biomarkers distinguishing trained and untrained horses.

## 2. Materials and Methods

### 2.1. Ethical Statement

This study was reviewed and approved by the Animal Policy and Welfare Committee of Xinjiang Agricultural University (Ethical Approval No.: 2023037). All experimental procedures strictly followed animal welfare and experimental ethics regulations. Informed consent was obtained from the horse owners. Before the experiment began, all horses underwent veterinary examinations and were confirmed to be in good health with no detected cardiac disease.

### 2.2. Experimental Design and Horse Grouping

Sixteen 18-month-old Yili horses (each group consisted of an equal number of male and female horses, from a state-owned stud farm in the Yili Kazakh Autonomous Prefecture, Urumqi, China) were selected for this study. The horses had similar birth dates with the same feeding and management conditions (For details, please refer to Supplementary Materials Table S1), minimal body size differences (the body measurement data are similar across groups. For details, please refer to Supplementary Materials Table S2), and were maintained under uniform feeding and management conditions. Ten horses underwent a three-month training program (trained group), while the remaining six were allowed free movement and were not exercised (untrained group). The jockey team at this facility is highly experienced, maintains a similar body weight, and demonstrates excellent professional competence.

### 2.3. Training Program

The trained group received the following training:

Week 1: desensitization training (both groups participated initially; however, only the training group continued after this week)

Desensitization phase:

1. Teach horse to listen with lead exercises;
2. Practice exercises to make horse comfortable with touch;
3. Train your horse to follow directions through circle work;
4. Introducing your horse to a saddle;
5. Place the saddle on your horse’s back;
6. Tighten the girth in intervals;
7. Use a mounting block to climb in the saddle;
8. Mount and dismount from the saddle in 10-min intervals.

Training period, starting from Week 5:

Begin walking your horse while in the saddle.

Week 2: thirty minutes in the lunge ring (walk—5 min, trot—20 min, and walk—5 min);

Week 3: forty minutes in the lunge ring (walk—5 min, trot—30 min, and walk—5 min);

Week 4: one hour on the horse walker (2 m/s—20 min, 3 m/s—30 min, and 4 m/s—10 min);

Week 5: thirty-five minutes of warm-up on the horse walker (2 m/s—5 min, 3 m/s—15 min, and 4 m/s—10 min), fifteen minutes of riding in the training arena (trot—5 minand canter—10 min);

Subsequent three weeks: an additional five minutes of riding (trot—5 min) each week;

Week 9: thirty-five minutes of warm-up on the horse walker (2 m/s—5 min, 3 m/s—15 min, and 4 m/s—15 min), fifteen minutes of track training (trot—5 minand canter—10 min, gallop);

Subsequent three weeks: An additional five minutes of track training per week until the 15th week, marking the end of the training program. (Weeks 10–12: based on Week 9, trot duration increased by 5 min per session. Weeks 13–14: canter duration increased by 5 min per session. Week 15: gallop duration increased by 5 min);

Trot at 50–60% HRmax, canter at 60–70% HRmax, and gallop at 70–80% HRmax.

### 2.4. Echocardiographic Evaluation

One day after the training ended, echocardiography was performed on the horses in a resting state using a Mindray M6 portable veterinary color Doppler ultrasound system. A 2.5 MHz probe was used for two-dimensional (2D) and M-mode imaging between the third and fourth or fourth and fifth ribs on the right thorax. The maximum imaging depth was set to 30 cm, with the transducer’s focal point fixed at 5 cm and a maximum sector angle of 110°. All echocardiographic examinations were conducted by the same operator, and images were recorded only when the heart rate was below 40 beats per minute. Three nonconsecutive cardiac cycles were measured, and the average value was taken.

A total of 22 cardiac parameters were measured and calculated from the right parasternal long-axis, right parasternal short-axis, and M-mode right parasternal short-axis images.

### 2.5. Quantitative Plasma Lipidomics Analysis by LC/MS-MS

Blood samples (resting state; 10 mL) were collected from the jugular vein of each horse into EDTA anticoagulation tubes. The samples were immediately centrifuged at 15,000× *g* for 15 min, and 1.5 mL aliquots of plasma were transferred into cryotubes, flash-frozen in liquid nitrogen, and stored at −80 °C for later lipidomics analysis.

1. Liquid chromatography column: Thermo Accucore™ C30 column (2.6 μm; 100 mm × 2.1 mm i.d.);
2. Mobile phases:
  Phase A: acetonitrile/water (60/40, *v*/*v*) containing 0.1% formic acid and 10 mmol/L ammonium formate;
  Phase B: acetonitrile/isopropanol (10/90, *v*/*v*) containing 0.1% formic acid and 10 mmol/L ammonium formate;
3. Gradient elution program:
  0 min: A/B = 80:20 (*v*/*v*);
  2 min: A/B = 70:30 (*v*/*v*);
  4 min: A/B = 40:60 (*v*/*v*);
  9 min: A/B = 15:85 (*v*/*v*);
  14 min: A/B = 10:90 (*v*/*v*);
  15.5 min: A/B = 5:95 (*v*/*v*);
  17.3 min: A/B = 5:95 (*v*/*v*);
  17.5 min: A/B = 80:20 (*v*/*v*);
  20 min: A/B = 80:20 (*v*/*v*);
4. Flow rate: 0.35 mL/min;
5. Column temperature: 45 °C;
6. Injection volume: 2 μL.

Lipid identification and quantification were based on the Metware self-built database, MWDB (https://www.metware.cn/, accessed on 29 January 2025). Lipids were identified by measuring their retention time and collecting parent–daughter ion pair data. Pearson correlation analysis was used to determine the relationship between differential metabolites and cardiac structure and function parameters, with the level for significance set at *p* < 0.05.

#### 2.5.1. Data Processing

Mass spectrometry data were processed using Analyst 1.6.3 software, and metabolites were identified and quantified using a local metabolic database. Detectable ions were screened using a triple quadrupole mass spectrometer, and their signal intensity (CPS) was recorded. Chromatographic peak integration and correction were performed using MultiQuant software 3.0.3. To ensure data reproducibility, a quality control (QC) sample was inserted after every ten samples. The final dataset was simplified and reduced through multivariate statistical analysis.

Principal component analysis (PCA) was conducted using the prcomp function in R software (version 3.5.1) with scale = true for unit variance scaling to identify metabolic differences between samples. Hierarchical clustering analysis (HCA) of metabolite data processed with unit variance scaling was performed using the ComplexHeatmap package in R, generating heatmaps to visualize the metabolic accumulation patterns across different samples.

Differential metabolites were screened using both univariate and multivariate analysis methods, including hypothesis testing, fold change (FC) analysis, and orthogonal partial least squares discriminant analysis (OPLS-DA). The selection criteria were VIP > 1 and *p* < 0.05, identifying metabolites significantly associated with training status.

#### 2.5.2. Screening of Differential Metabolites and KEGG Functional Analysis

The identified differential metabolites were functionally annotated using the KEGG database to identify associated biological pathways. Volcano plots were generated using the ggplot2 package in R to visualize significantly different metabolites between groups, while the pheatmap package was used to generate heatmaps showing metabolite clustering across samples. KEGG enrichment analysis was conducted to assess pathway significance, with a screening threshold of *p* < 0.05, identifying significantly enriched metabolic pathways.

#### 2.5.3. Correlation Analysis Between Cardiac Structure, Function, and Plasma Lipid Composition

Pearson correlation analysis in SPSS 26.0 was used to assess the relationships between cardiac structure and function indices and 153 differential lipid metabolites with VIP > 1.5. The significance thresholds were set at *p* < 0.05 (*) for significant correlations, *p* < 0.01 (**) for highly significant correlations, and *p* < 0.001 (***) for very highly significant correlations.

### 2.6. Statistical Analysis

All figures were generated using GraphPad Prism 8.0 (GraphPad Software Inc., San Diego, CA, USA). Statistical analyses included analysis of variance (ANOVA), conducted using SPSS 26.0 (IBM, Armonk, NY, USA). Data are expressed as mean ± standard deviation, and group differences were assessed using one-way ANOVA. Homogeneity of variance within groups was tested, with *p* > 0.05 indicating no significant variance difference.

## 3. Results

### 3.1. Analysis of Differences in Cardiac Structure Between Trained and Untrained Yili Horses

**Figure 1:**
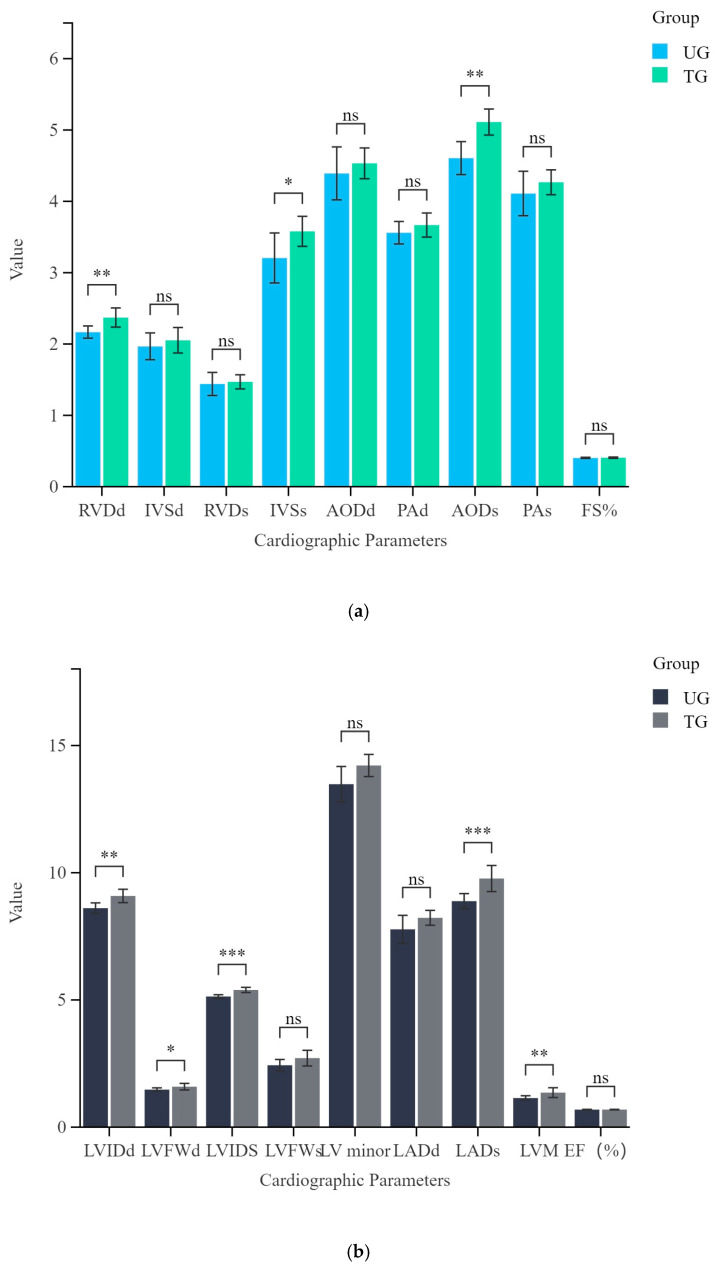
Statistical comparisons of echocardiography results from the two groups(UG and TG) of Yili horses. (**a**) Differences of RVDd, IVSd, RVDs, IVSs, AODd, PAd, AODs, PAs, FS% between UG group and TG group. (**b**) Differences of LVIDd, LVFWd, LVIDs, LVFWs, LV minor, LADd, LADs, LVM, EF% between UG group and TG group. ns: not significant, * *p* < 0.05, ** *p* < 0.01, and *** *p* < 0.001.

This study included 16 Yili horses, with 10 horses undergoing three months of training compared with 6 untrained horses moving freely in the activity field under the same feeding and management conditions. Table 1 summarizes the cardiac structure and functional characteristics of all horses. Figure 1a shows there are no significant differences in ejection fraction (EF%) and fractional shortening (FS%) between the trained and untrained groups. However, significant differences were observed in other cardiac structural and functional parameters (Figure 1a,b).

### 3.2. Changes in Quantitative Lipidomics Between Trained and Untrained Yili Horses

#### 3.2.1. Identification of Differentially Expressed Lipid Metabolites in Plasma

**Figure 2:**
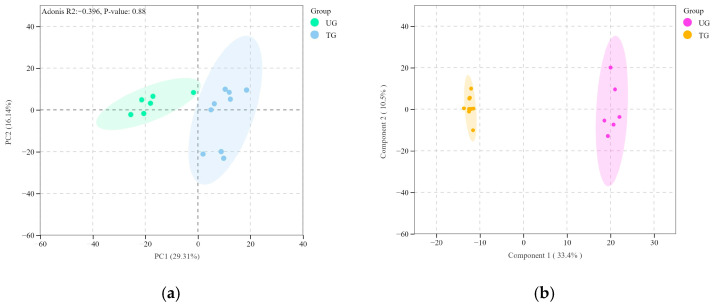
Quality control analysis of equine plasma in UG versus TG. (**a**) Plot of principal component analysis. (**b**) Map of OPLS-DA scores.

Using lipidomics technology and UPLC-MS/MS quantitative data, 347 metabolites were identified. Principal component analysis (PCA) was performed on all experimental samples and quality control (QC) samples to preliminarily assess the degree of variation between samples (Figure 2a). The analysis revealed differences among samples, with distinct separation observed between the trained group (TG) and the untrained group (UG). All samples fell within the elliptical confidence interval, indicating clear differentiation between the two groups.

Orthogonal partial least squares discriminant analysis (OPLS-DA) was used to analyze the lipid metabolites in the plasma of Yili horses before and after training. The score plot showed a clear separation between the trained and untrained Yili horses after three months of training, demonstrating significant changes in plasma lipid metabolites (Figure 2b).

#### 3.2.2. Analysis of Differentially Expressed Lipid Metabolites in Plasma

**Figure 3:**
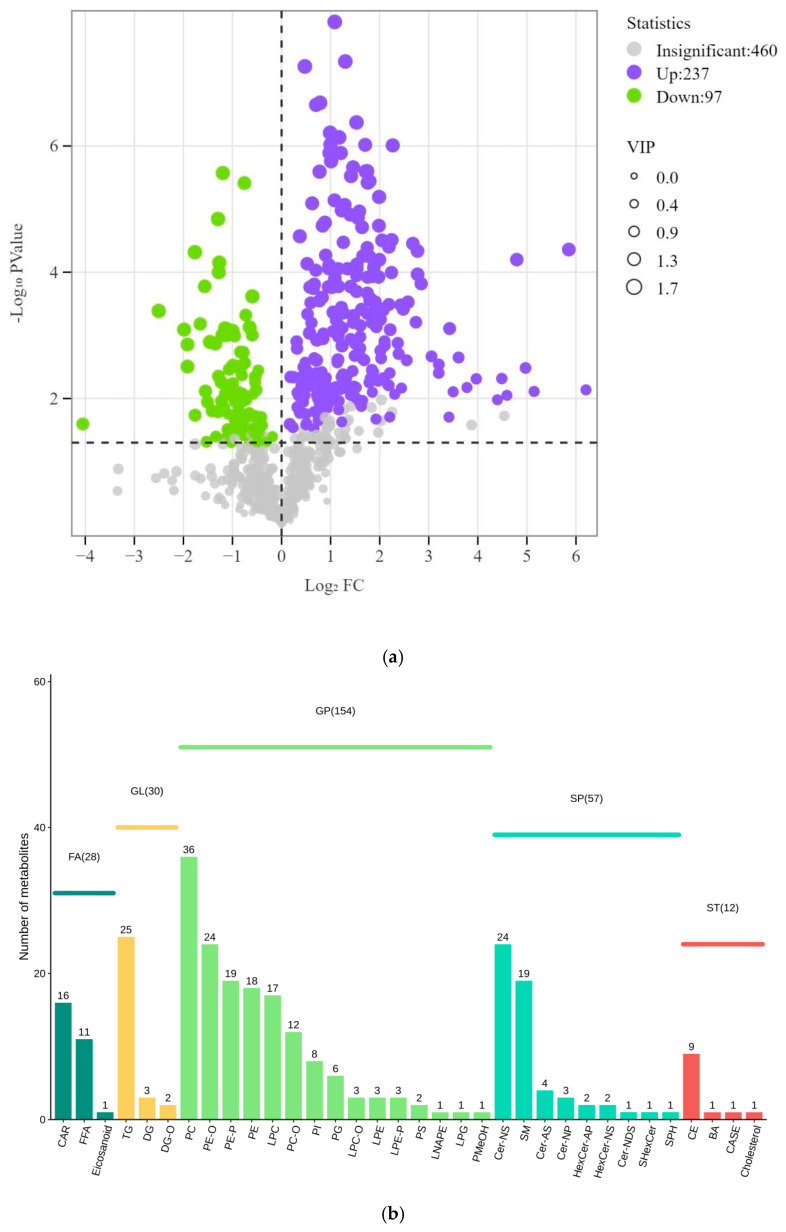
Metabolome analysis of equine plasma in UG and TG. (**a**) Volcano plot of differential lipids between trained and untrained Yili horses. (**b**) Bar chart of the number of differential lipid subclasses between trained and untrained Yili horses.

To gain a clearer understanding of the lipid metabolic changes between trained and untrained Yili horses, we used an absolute quantitative lipidomics approach to identify lipid subclasses and lipid molecules in the samples. A total of 34 lipid subclasses and 281 lipid molecules were detected in horse plasma (Figure 3a). Compared with untrained Yili horses, 212 lipids were upregulated, and 69 lipids were downregulated in the trained group. This indicates that exercise training plays a crucial role in the regulation of lipid metabolism, suggesting that it could be used for improving lipid metabolic health. Interestingly, although a larger number of lipid metabolites were classified under the glycerophospholipid (GP) category, the triacylglycerol (TG) subclass within the glycerolipid (GL) category showed a numerical advantage (Figure 3b).

#### 3.2.3. Pathway Enrichment Analysis of Differential Lipid Metabolites

**Figure 4:**
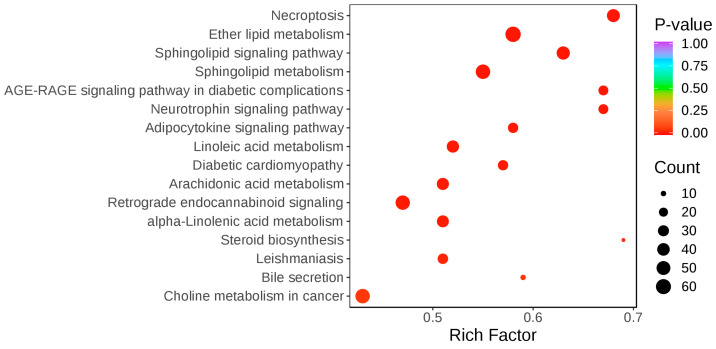
KEGG enrichment bubble plot of differential lipids between the trained and untrained groups. *p*-value: significance test *p*-value; count: number of lipids enriched in the pathway; rich factor: enrichment factor, representing the ratio of the number of differential lipids annotated to a given pathway to the total number of lipids annotated to that pathway. A higher rich factor indicates a greater degree of enrichment.

All differential lipid metabolites between trained and untrained Yili horses were mapped to the KEGG database to obtain information on the metabolic pathways in which these metabolites participate. An enrichment analysis was performed on the annotated results to identify pathways with the highest enrichment of differential metabolites, and a KEGG enrichment bubble plot was generated (Figure 4). The differential lipids between trained and untrained Yili horses were primarily annotated and enriched in pathways such as necroptosis, ether lipid metabolism, and the sphingolipid signaling pathway, which are involved in cell migration, survival, and proliferation. Additionally, significant enrichment was observed in pathways related to linoleic acid metabolism, arachidonic acid metabolism, and alpha-linolenic acid metabolism.

#### 3.2.4. Correlation Analysis Between Differential Metabolites and Cardiac Structure

**Figure 5:**
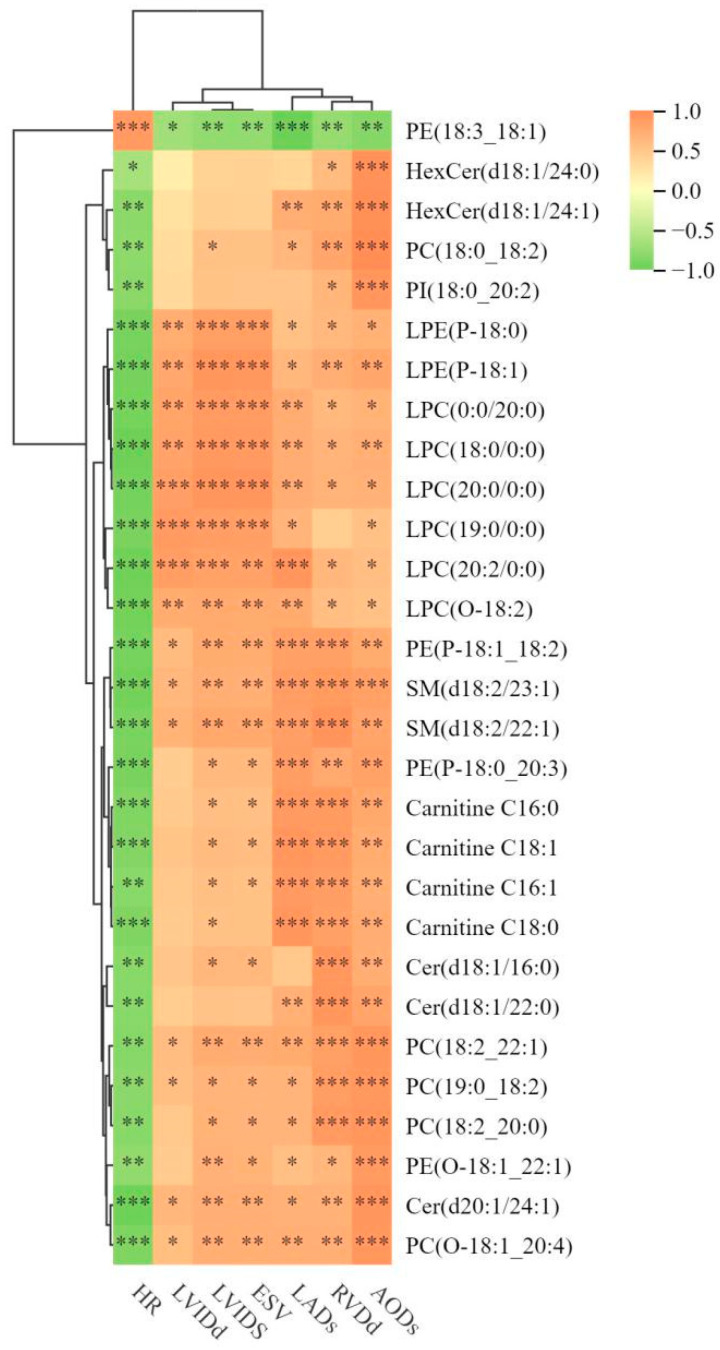
Correlation between lipid metabolites and cardiac structure and function. * *p* < 0.05, ** *p* < 0.01, and *** *p* < 0.001. Orange indicates a positive correlation, while green indicates a negative correlation.

As shown in Figure 5, the variable importance in projection (VIP) value represents the influence strength of each lipid’s intergroup differences in classifying samples within the model. A higher VIP value indicates that the corresponding lipid contributes more significantly to the differentiation between the two groups. By performing Pearson correlation analysis between key metabolites with high VIP values and cardiac structure and function, we identified significant correlations between them.

## 4. Discussion

Cardiac remodeling refers to compensatory or decompensatory changes in cardiac genes, proteins, cells, and the intercellular matrix, which clinically manifest as structural and functional alterations of the heart [16]. Lipid metabolism is an indispensable component of cardiac remodeling, as lipids serve as the major energy source for the heart and are influenced by multiple factors [17]. This study compared the differences in cardiac structure, function, and absolute quantitative lipids in the plasma between trained and untrained Yili horses, providing new insights into the molecular mechanisms of physiological cardiac remodeling induced by long-term training.

The Yili horse is a breed native to the Xinjiang Uygur Autonomous Region of China. They combine the advantages of their Kazakh mare lineage, such as the ability to digest rough forage and strong disease resistance, with the speed and endurance of Thoroughbred sires. Although the statistics are incomplete, more than 16,000 Yili horses with athletic potential have been registered with the Xinjiang Horse Industry Association (https://horse.xjau.edu.cn/, accessed on 29 January 2025).

The heart of a horse accounts for approximately 0.9% to 1% of its total body mass, and in systematically trained horses, heart mass can reach 1.1% of total body weight [18,19]. The left ventricle (LV) is the primary pumping chamber, increasing myocardial contractility and cardiac output during exercise to meet the body’s oxygen and nutrient demands. After prolonged racing and systematic training, myocardial cells enlarge, leading to adaptive left ventricular hypertrophy, which significantly increases total heart mass [4,5,20]. Our echocardiographic measurements revealed that LVIDd, LVIDs, LVFWd, and IVSs were significantly greater in the trained group compared to the untrained group, while LVFWs, IVSd, and LV minor showed no statistical difference but were still larger in the trained group. A review of exercise-induced cardiac remodeling concluded that a wealth of literature supported the notion that cardiac remodeling was a widespread phenomenon, with the distinctive characteristics of an “athlete’s heart” observed in humans, horses, and dogs [1]. At rest, trained horses had significantly lower heart rates than untrained horses, while EDV, ESV, SV, CO, and LVM were significantly higher, indicating that three months of incremental training contributes to adaptive cardiac remodeling.

Most studies on equine cardiac remodeling focused on the left ventricle due to its crucial role in maintaining cardiac output during exercise. However, recent research has revealed a dynamic interdependence between the right and left ventricles. The right ventricle supports left ventricular filling by increasing end-diastolic volume, thereby maintaining overall cardiac pump function [21]. Another study found that different athletic disciplines only influenced right ventricular structure in 35% of athletes, with changes being non-significant [22]. In this study, RVDd was significantly increased in the trained group compared to the untrained group, while RVDs showed no significant changes, suggesting that longer training durations may be required for substantial right ventricular adaptation. The aorta plays a crucial role in buffering pulsatile cardiac output to reduce the risk of vascular damage due to blood pressure fluctuations [23]. Long-term training induces adaptive changes in the aorta, which contribute to cardiovascular health [24]. In this study, AODs was significantly larger in the trained group, whereas AODd did not follow the same trend, indicating that training positively stimulated aortic structural adaptation in Yili horses.

The relationship between lipids and physiological cardiac remodeling is complex and multifaceted. Lipids play a key role in cardiac metabolism and energy production, and their dysregulation can significantly alter cardiac function and structure. In exercise-induced cardiac remodeling, lipids contribute to metabolic adaptations that support increased cardiac workload capability and efficiency [25].

Phosphatidylcholine (PC) and phosphatidylethanolamine (PE) are the most abundant phospholipids in cell membranes. PC not only maintains mitochondrial membrane integrity but also regulates mitochondrial bioenergetics, supporting efficient electron transport chain function [26]. Another study found that the peak values of PC (20:4/18:0) and PE (20:4/18:0) were negatively correlated with the heart weight/body weight ratio, suggesting that these lipids may have protective roles in cardiac remodeling [27]. Lysophosphatidylcholine (LPC), a hydrolysis product of PC, has important biological functions. LPC is involved in the pathogenesis of cardiovascular diseases, including atherosclerosis and inflammatory responses [28]. In this study, PC (17:0_18:1) (VIP = 1.98) and PE (20:4_18:0) (VIP = 2.01) showed significant differences between the two groups and strong positive correlations with RVDd and AODs. Additionally, LPC subclasses (LPC (18:0/0:0), LPC (0:0/15:0), LPC (15:0/0:0), and LPC (20:0/0:0)) were significantly correlated with LVID, further emphasizing the role of lipid metabolism in cardiac structural adaptations.

Exercise can regulate lipid metabolism by influencing intracellular ceramide (Cer) levels, which are essential for energy balance and metabolic homeostasis. [29]. Ceramide plays a key role in muscle energy metabolism, and its level is correlated with metabolic state and energy demand. Elevated ceramide levels can increase muscle cell apoptosis, affecting muscle mass and function [30]. This study found that Cer (d20:1/24:1) was more abundant in the trained group and significantly positively correlated with AODs.

The differential lipids between trained and untrained horses were enriched in the sphingolipid signaling pathway. Sphingomyelin (SM) was not only widely present in neuronal cell membranes but also regulates signal transduction and cellular metabolism. Studies have shown that sphingomyelin influenced membrane protein function and interactions, playing a key role in neuronal signaling and intercellular communication [31,32]. SM (d40:4) was primarily enriched in the sphingolipid signaling pathway, which has been shown to be essential for myocardial proliferation and cardiac regeneration [33]. Linoleic acid metabolism is vital for cardiovascular health, as linoleic acid derivatives regulate inflammation and vascular function, while arachidonic acid metabolism influences inflammatory responses, vascular tone, and platelet aggregation [34,35]. These enrichments of metabolic and signaling pathways demonstrate that intensive exercise training can significantly enhance cardiac energy metabolism, structural adaptation, and functional optimization. This study provides new theoretical support for understanding the adaptive mechanisms of exercise training on cardiac function and for designing more effective exercise regimens.

## 5. Conclusions

In summary, the differential plasma lipids PE (20:4_18:0), PC (17:0_18:1), and LPC subclasses in trained and untrained Yili horses were significantly correlated with cardiac structure and function. The trained group exhibited more pronounced cardiac remodeling and metabolic regulation advantages, including enhanced metabolic adaptation and structure–function optimization through pathways such as the sphingolipid signaling pathway, linoleic acid metabolism, and arachidonic acid metabolism. This study integrated cardiac structure, function, and lipid metabolism to evaluate the differences between trained and untrained Yili horses, potentially providing insights into more effective training strategies and cardiac regulation mechanisms for sport horses.

## Figures and Tables

**Table 1:** Results of echocardiography of the two groups of Yili horses under resting conditions showing cardiac structural and functional measurements.

TERM	Untrained Group (UG)	Trained Group (TG)
RVDd (cm)	2.17 ± 0.09	2.37 ± 0.13
IVSd (cm)	1.97 ± 0.19	2.05 ± 0.18
LVIDd (cm)	8.61 ± 0.21	9.09 ± 0.26
LVFWd (cm)	1.48 ± 0.07	1.59 ± 0.13
RVDs (cm)	1.44 ± 0.16	1.47 ± 0.1
IVSs (cm)	3.21 ± 0.35	3.58 ± 0.21
LVIDs (cm)	5.14 ± 0.07	5.39 ± 0.1
LVFWs (cm)	2.43 ± 0.22	2.71 ± 0.31
LV minor (cm)	13.48 ± 0.7	14.22 ± 0.43
LADd (cm)	7.78 ± 0.55	8.23 ± 0.29
LADs (cm)	8.88 ± 0.3	9.77 ± 0.51
AODd (cm)	4.39 ± 0.37	4.53 ± 0.22
PAd (cm)	3.56 ± 0.16	3.67 ± 0.17
AODs (cm)	4.61 ± 0.23	5.11 ± 0.18
PAs (cm)	4.11 ± 0.31	4.27 ± 0.17
EF (%)	0.69 ± 0.01	0.69 ± 0.01
FS%	0.4 ± 0.01	0.41 ± 0.01
LVM (kg)	1.14 ± 0.09	1.36 ± 0.19
EDV (mL)	405.77 ± 21.99	457.75 ± 29.38
ESV (mL)	125.85 ± 3.96	140.92 ± 6.41
SV (mL)	279.92 ± 18.39	316.83 ± 23.98
HR	45.03 ± 2.24	43.93 ± 3.96
CO (L/min)	15.42 + 1.41	13.89 + 1.27

Note: UG, untrained control group, *n* = 6; TG, trained group, *n* = 10; RVDd: end-diastolic right ventricular diameter; IVSd: end-diastolic interventricular septal thickness; LVIDd: end-diastolic left ventricular diameter; LVFWd: end-diastolic left ventricular free wall thickness; LADd: end-diastolic left atrial diameter; LADs: end-systolic left atrial diameter; AODd: end-diastolic aortic root diameter; PADd: end-diastolic pulmonary artery diameter; PADs: end-systolic pulmonary artery diameter; EF: ejection fraction; FS: fractional shortening; LVminor: left ventricular minor; SV: stroke volume; EDV: end-diastolic left ventricular volume; ESV: end-systolic left ventricular volume; CO: cardiac output; LVM: left ventricular myocardial mass; HR: heart rate. Data are reported as mean + SD for all variables.

## Data Availability

The data supporting this study’s findings are available from the corresponding author upon reasonable request.

## Supplementary Materials

The following supporting information can be downloaded at https://www.mdpi.com/article/10.3390/vetsci12030255/s1: Table S1: Composition and nutritional levels of the basal diet (on dry matter basis); Table S2: Body measurements of horses before training.
