# Supplementary material for: Absolute Quantitative Lipidomics Reveals Differences in Lipid Compounds in the Blood of Trained and Untrained Yili Horses
Source: Vet Sci. 2025 Mar 10;12(3):255. doi: 10.3390/vetsci12030255 (PMC11945474; doi:10.3390/vetsci12030255)
Supplement: Supplementary file 1 [file vetsci-12-00255-s001.zip › vetsci-3484355-supplementary.pdf]

Supplementary Materials

Each horse was fed 8 kg/d of dry forage and 4 kg/d of concentrate supplement.

Table S1. Composition and nutritional levels of the basal diet (on dry matter basis).

Item	Content (%)
**Ingredients**	
Corn	17.28
Wheat bran	5.26
Soybean meal	9.26
Monocalcium phosphate	1.15
Salt	0.63
Premix	0.31
Methionine	0.19
Dry forage	65.92
Total	100
**Nutritional Levels**	
Dry matter	95.31
Crude protein	12.69
Crude fat	1.77
Neutral detergent fiber	42.23
Acid detergent fiber	37.98
Crude ash	8.16
Calcium	0.86
Phosphorus	0.41
**Digestible energy (MJ/kg)**	9.03

Note: 1. The premix provided the following per kg of the concentrate supplement: VA 14 mg, VB1 21.29 mg, VB2 336.5 mg, VB6 1.27 mg, VD 2.3 mg, VE 857 mg, biotin 6 mg, pantothenicacid 4.56 mg, nicotinamide 12.17 mg, Cu (as copper sulfate) 43.24 mg, Fe (as ferrous sulfate) 111.94 mg, Mn (as manganese sulfate) 183.27 mg, Zn (as zinc sulfate) 176.04 mg, I (as potassium iodide) 29.69 mg, Se (as sodium selenite) 42.29 mg, Co (as cobalt chloride) 4.06 mg.

2. Nutrient levels were measured values.

Table S2. Body measurements of horses before training.

Index	Weight	Height	Body length	Chest measurement	Perimeter
UGA1	328.58	149.00	142.00	161.00	17.00
UGA2	309.45	145.00	139.00	157.00	17.00
UGA3	312.38	145.5	139	158	17
UGA4	304.05	144	138	156	17
UGA5	323.35	145	147	155	16.5
UGA6	341.39	147	146	162	18
TGA1	323.64	143	140	161	17
TGA2	322.72	143	142	159	17

TGA3	332.60	150	146	159	16.5
TGA4	343.40	152	148	161	17
TGA5	328.58	146	142	161	17.5
TGA6	336.45	147	144	162	17
TGA7	310.37	142	137	159	17.5
TGA8	338.46	147	146	161	17
TGA9	341.39	146	146	162	17.5
TGA10	359.14	151	152	163	17
